# Foreign Correspondence

**Published:** 1869-03

**Authors:** 


### Foreign Correspondence.

VIENNA, *December 25th, 1868.*

DEAR EXAMINER:—I once met a man travelling through the State of Illinois in search of pine forests. He had found a few scattered trees on the banks of some of its streams; and was so encouraged that he continued his search. Not less amusing or humiliating is it to find comparatively intelligent American physicians in Europe studying particular branches of medicine, in cities where these branches are poorly taught and illustrated. For instance, one meets Americans in Wurtzburg paying especial attention to skin disease, where but little is found; instead of going to Hardee, at Paris, or Hebra, in this city, who have the most abundant material. Others stop a year at Heidelberg, to learn midwifery, when they could, perhaps, see more at Prague in a month: while many others cross the sea and study topics they had far better learn at home.

European students often think we are entirely ignorant of their institutions; and often, not without just reason. I was told in America that I would be allowed here to make all the operations in midwifery, and perform many important operations in surgery. On the contrary, I am permitted to make no considerable operation in the latter, and only to apply the forceps in the former.

I have thought that, perhaps, I might communicate something, both to those of your readers who may chance to visit Europe, for the purpose of study, and for the intelligence of those who remain at home, on Vienna as a place for the study of medicine; which might be of more benefit for the present than the detail of special cases; not intending, however, to compare this with other European hospitals or schools, but to state, in a simple manner, what opportunities the student may find here; leaving it for the reader to make the comparison with what he may know of other places. It is scarcely necessary that I should speak of the medical faculty. The names of Skoda,

Rokitansky, Sigmund, Hebra, Oppolzer, etc., are familiar to every medical scholar. I may hereafter speak of their treatment of special cases; which will better illustrate the worth of the men than anything I can otherwise say of them.

The Allgemeineu Kraukenham (General Hospital) has, at present, somewhat over two thousand patients in its wards; while between one and two hundred out-patients visit its clinics daily. The hospital building is in the form of a hollow square, with cross-sections, which divide the square into nine or ten smaller courts; so that only a minute or two is necessary to go from one clinic to another; which are so arranged, in regard to time, that one is enabled to attend from six to ten clinics daily.

I shall first speak of the department of obstetrics and diseases of women, and how they are studied. About 10,000 women are delivered in the two lying-in wards annually. In one of these wards the material is used by Professor Späth, for the instruction of midwives; the other is accessible to students of general medicine, male and female. Adjoining the ward is an amphitheatre, where Professor Braun delivers a clinical lecture, five times a week. Difficult cases are brought in, and delivered, on the table before the class, by the forceps, version, craniotomy, etc., as the case may require. Students are sometimes called upon to apply the forceps. I may here state that a female student (Russian), though strong and vigorous, has been entirely unable, through want of strength, in one or two cases, not very difficult, to extract with the forceps—if it be any argument against female practitioners? Women demanding surgical treatment are also operated on before the class. After the clinic, we often visit, for a few minutes, the gynæcological ward, of fourteen beds, and see a little of the treatment of female disease. Twelve students of the class are allowed to remain in the ward every twenty-four hours, and deliver normal cases, under the direction of the midwives; and those who have taken private courses in operative midwifery, of either of the Assistants, are often allowed to apply the forceps, and make traction, under the direction of an Assistant or the Professor; but to make no other operation. Neither students nor midwives are allowed to make any operation without the presence of an Assistant or Professor, however urgent the case. This results in some evil as well as good. I've seen some cases lie for two hours, and finally result in eclampsia, because no Assistant could be found, and no student or graduated physician allowed to make some simple operation, as applying the forceps, which might have avoided this terrible calamity.

Private courses of operative midwifery are given by the Assistants, Drs. Rokitansky, Jr., and Mayerhofer, in which each student may make all operations on the cadaver of mother and child. Only those who have taken this course are allowed to apply the forceps in the ward.

On four evenings of the week, pregnant women, desiring to enter the lying-in room, are examined for admission. Those who have passed the seventh month are admitted. Students who wish are allowed to examine these cases, by palpation, and per vaginam, to determine the position of the fœtus, stage of pregnancy, condition of the organs, etc. We have no instruction in this, except the casual remarks made by the Assistant, as he passes from one case to another. The number of cases varies from one to ten; generally five or six. The other three evenings they are examined in Professor Späth's wards, to which, as I've before remarked, we are not admitted.

Although there appears to be abundant material to illustrate the diseases of women, over forty cases, yet there is no special Professor to illustrate this department. With the exception of the few cases in Professor Braun's ward, above mentioned, this department is in charge of a hospital physician, not professor, whose assistant gives private courses to classes of ten, in diagnosis and treatment of female diseases. Each student is allowed to examine cases, and give his diagnosis, which is confirmed or corrected according to circumstances; also, to introduce sound, catheter, and speculum. Our instruction, however, is not the most scientific or professional; and, I should judge, the student would be far more successful in practice by following the instruction of Byford or Thomas, in these cases, than by adhering to precepts here received. In this private course I've seen no pessaries used, or operation performed for the displacement of the uterus. Uterine and vaginal discharges are not carefully distinguished and classified; hence, probably, not the most successfully treated.

I may add, that patients are generally removed from the lying-in wards from one to three hours after delivery, and are seldom bandaged. Cases of eclampsia are treated with large doses of morphine; rarely bled, or given chloroform. Medicine is rarely used to prevent secretion of milk. They claim equally good results, in both these departments, with other hospitals; but, if I mistake not, it depends largely on the class of patients, many of whom are sturdy peasants from the country, and can scarcely be compared with the hospital patients of Chicago, much less with those of private practice.

I shall endeavor to present some statistics of the hospital, on these points, at a subsequent writing. F.

